# Association of haptoglobin and natural resistance-associated macrophage protein 1 alleles with heme-consuming periodontal pathogens in chronic periodontitis and peri-implantitis: A pilot study

**DOI:** 10.34172/japid.2020.006

**Published:** 2020-05-02

**Authors:** Mahdi Kadkhodazadeh, Ahmad Reza Ebadian, Zahra Alizadeh Tabari, Reza Amid, Anahita Moscowchi

**Affiliations:** ^1^Dental Research Center, Shahid Beheshti University of Medical Sciences, Tehran, Iran; ^2^Private Practice, Tehran, Iran; ^3^Department of periodontics, International Campus, School of Dentistry, Tehran University of Medical Sciences, Tehran, Iran; ^4^Department of Periodontics, Dental School, Shahid Beheshti University of Medical Sciences, Tehran, Iran

**Keywords:** Chronic Periodontitis, Haptoglobin, Microorganism, NRAMP1, Pathogens, Peri-implantitis

## Abstract

**Background:**

This study aimed to assess the association of haptoglobin (HP) and natural resistance-associated macrophage protein 1 (NRAMP1) alleles with the presence of heme-consuming periodontal pathogens in a group of Iranian patients with chronic periodontitis and peri-implantitis.

**Methods:**

This cross-sectional study evaluated 69 eligible chronic periodontitis and peri-implantitis patients selected from Shahid Beheshti Dental School. The periodontally diseased individuals had at least three teeth with clinical attachment loss of ≥3 mm and a probing pocket depth (PPD) of ≥3 mm in at least two quadrants. Peri-implant PPD of at least one site was ≥5 mm with or without suppuration and bleeding on probing. A plaque index of >20% and a radiographic crestal bone loss was present in at least one site around the implant. The paper point method was used for sampling from the deepest periodontal/peri-implant pocket of each tooth or implant for the DNA checkerboard hybridization technique. Statistical analyses were performed with PASW Statistics 18.0. The variables were presented as absolute and relative frequencies (%).

**Results:**

An Aggregatibacter actinomycetemcomitans (A. actinomycetemcomitans) score of 1–2 was 5.8 times more frequent in HP 2, rs1723540 G, and rs2276631 G alleles. A Porphyromonas gingivalis (P. gingivalis) score of 1–2 was 4.8 times more common in the subjects carrying HP 2, rs1723540 G, and rs2276631 G alleles compared with HP 1, rs1723540 A, and rs2276631 A alleles.

**Conclusion:**

Within the limitations of this study, it seems that there was a relationship between HP and NRAMP1 allele frequencies and the presence of heme-consuming periodontal pathogens in the Iranian patients with chronic periodontitis and peri-implantitis evaluated in the present study.

## Introduction

Chronic inflammatory diseases of the tooth-supporting structures are often associated with bone loss. The underlying mechanisms of chronic inflammatory diseases are complex and interrelated. Although inflammatory diseases can increase bone resorption and decrease bone formation, they most commonly affect both of these mechanisms.^1^ Periodontitis and peri-implantitis are multifactorial inflammatory diseases caused by the activity of periodontal pathogens and the host inflammatory responses. The imbalance between these two factors results in connective tissue attachment loss and alveolar bone destruction.^2^ Although the pathogenic gram-negative bacteria play a critical role in periodontitis and peri-implantitis, the onset and progression of these diseases are affected by a combination of environmental and host-related factors.^3^

Free hemoglobin (Hb) and heme might meet the bacterial requirement for iron, and some bacteria such as black-pigmented *Bacteroides* including *Porphyromonas gingivalis (P. gingivalis)*, *Prevotella intermedia (P. intermedia), P. nigrescens*, and other periodontal pathogens, such as *Aggregatibacter actinomycetemcomitans (A. actinomycetemcomitans)*, *Treponema denticola (T. denticola),* and *Campylobacter rectus (C. rectus)* consume iron as an essential nutrient. The availability of ionic iron or iron-containing compounds might affect bacterial growth in tissues; however, some proteins such as haptoglobin (HP) and natural resistance-associated macrophage protein 1 (NRAMP 1) might play essential roles in the host defense mechanisms against bacterial infections by depriving the tissue-invading bacteria of nutritional iron.^4,5^

HP is an acute phase protein produced primarily in the liver, which increases in response to inflammatory stimuli. HP has several biological functions, but it is best known as an Hb-binding protein. Therefore, HP, as a heme scavenging protein, seems to have a bacteriostatic effect on heme-consuming bacteria.^6^ The HP gene is located on chromosome 16q22 and has two major classes of functional alleles in humans. These alleles, known as 1 and 2, can form three different homozygous (1-1 or 2-2) and heterozygous (2-1) genotypes.^7^ These genotypes have different Hb-binding affinities. People carrying the 1-1 genotype have the highest binding affinity, while some inflammatory diseases, such as atherosclerosis, inflammatory bowel disease, celiac disease, and diabetes mellitus, are more prevalent in patients carrying the 2-2 genotype.^8-11^

NRAMP1 has considerable effects on the macrophage function (phagocytosis) and the host innate immune response against infections. NRAMP1 is also a heme-binding agent and plays a bacteriostatic role in infections caused by bacteria for which heme is an essential nutrient.^5^ NRAMP1 is encoded by the solute carrier family 11a member 1 (SLC11A1) gene. The location of the SLC11A1 gene is on the chromosome 2q35, and it has 15 exons spanning about 14 Kb.^12,13^

The SLC11A1 has several single nucleotide polymorphisms (SNPs), including rs1723540 (A/G) (in exon) and rs2276631 (A/G) (in intron) that change the protein function.^14^ Many studies have evaluated the association of these SNPs with inflammatory, autoimmune, and infectious diseases, including visceral leishmaniasis, tuberculosis, inflammatory bowel disease, multiple sclerosis, leprosy, type 1 diabetes mellitus, Crohn’s disease, and rheumatoid arthritis.^12-14^

The DNA checkerboard is a technique for simultaneous and quantitative analysis of up to 28 plaque samples for 40 microbial species.^16^ It was initially developed to predominantly study the gram-negative subgingival microorganisms involved in periodontitis. The DNA checkerboard technique offers the ability to include more potential periodontal pathogens in large-scale studies with a single analysis that is usually plausible with culture-based analysis.^17^

To date, only one study has evaluated genotype and allele frequencies of HP and NRAMP1 polymorphisms in patients with periodontitis and peri-implantitis. It has been reported that rs17235409 and rs2276631 polymorphisms (A to G allele substitution in the NRAMP1 gene) were associated with chronic periodontitis in an Iranian population.^18^ However, no significant differences were found between different genotypes and allele frequencies of HP in patients and healthy controls.^19^

To the best of the authors’ knowledge, no previous study has evaluated the relationship of the above gene polymorphisms and periodontal and peri-implant pathogens. Since heme-consuming pathogens are related to the most adverse outcomes in periodontal and peri-implant diseases, detection of a potential relationship between their presence and the candidate alleles might result in promising improvements in the early diagnosis and more successful management of these conditions. Therefore, the current study aimed to assess the association of HP and NRAMP1 allele frequencies with the presence of heme-consuming periodontal pathogens (using the DNA checkerboard hybridization technique) in a group of Iranian patients with chronic periodontitis and peri-implantitis.

## Methods

In this cross-sectional study, 69 chronic periodontitis and peri-implantitis patients who met the inclusion criteria were selected among those referred to the Department of Periodontics, Shahid Beheshti University of Medical Sciences. The subjects filled out the demographic, medical, and dental history questionnaires, and written informed consent was obtained from them. The study was approved by the Ethics Committee of Shahid Beheshti University of Medical Science (Tehran, Iran, Ref. no. # IR.SBMU.DRC.REC.1398.164).

The exclusion criteria consisted of oral diseases other than caries and periodontal diseases, ongoing orthodontic therapy, a history of systemic or local diseases affecting the immune system, diabetes mellitus, hepatitis, HIV infection, immunosuppressive chemotherapy, pregnancy or lactation, and non-Iranian ethnicity.

The patients in the chronic periodontitis group had at least five teeth, except for the third molars in each quadrant. A diagnosis of chronic periodontitis was reached based on radiographic and clinical parameters, including plaque index,^20^ probing pocket depth (PPD), clinical attachment loss, and bleeding index.^21^ The measurements were made at four points around each tooth by an experienced periodontist. Periodontally diseased individuals had at least three teeth with clinical attachment loss of ≥ 3 mm and PPD of ≥3 mm in at least two quadrants. Patients in the peri-implantitis group had one or more implants that were functionally loaded for more than one year. Peri-implant PPD of at least one site was ≥5 mm with or without suppuration/bleeding on probing. A plaque index of >20% and radiographic crestal bone loss were present in at least one site around the implant, resulting in the exposure of at least two implant threads. Patients who were in class VI, VII, and VIII of the Implant Success Index were enrolled in this study.^22^

In this study, the blood samples (5 mL) of the patients, obtained from their cephalic vein for another study, were used to determine the effect of osteoprotegerin gene polymorphism conducted by the same group of authors.^23^ The DNA was extracted using the DNA extraction kit (CinnaGen Inc., Tehran, Iran) according to the manufacturer’s instructions. For the genotyping of NRAMP1, the DNA samples were re-extracted and sent to KBioscience Ltd Co. (Hoddesdon, UK) since they perform the genotyping of SNPs that have only two conditions (A/G, etc.).

HP has three genotype conditions (1.1, 2.2, and 2.1). The genotyping of HP was conducted in the Cellular and Biomolecular Center of Shahid Beheshti Dental School. The genotyping process has been thoroughly explained in a previous study.^19^

There are three possible Hp genotypes: 1.1, 1.2, or 2.2. These genotypes have different Hb binding affinities. People carrying the 1.1 genotype have the highest binding affinity, and those carrying the 2.2 genotype have the lowest.

### Bacterial sampling and analysis

The paper point method was used for sampling from the deepest periodontal/peri-implant pocket around each tooth or implant. A sterilized medium-size paper point (#40; T.g., UK) was inserted into the bottom of the deepest pocket site and kept in place for 15–20 seconds. The soaked paper points were placed in Eppendorf tubes and sent to the Oral Microbiology Laboratory, Institute of Odontology, Sahlgrenska Academy at University of Gothenburg, Sweden, to quantify the following bacterial species: *P. gingivalis, P. intermedia, P. nigrescens, T. denticola, A. actinomycetemcomitans,*and* C. rectus*, using DNA checkerboard hybridization technique according to the protocol described in a previous study.^24^

### Statistical analysis

Statistical analyses were performed with PASW Statistics 18.0 (SPSS Inc., Chicago, IL, USA). The variables were presented as absolute and relative frequencies (%).

## Results

Due to the limited sample size in the present study and for better understanding, some data had to be merged. The 1–5 scoring system of DNA checkerboard hybridization analysis (Tables 1–3) was reduced to two categories of a: 1–2 and b: 3–5.

**Table 1:** Frequencies of iron-dependent bacteria in HP and NRAMP1-rs1723540 alleles

**HP and rs1723540**		**Allele 2, G**	**Allele 1, A**	**Total**
**P. gingivalis**	Score 1, 2	123	27	150
	Score 3, 4, 5	30	6	36
**P. intermedia**	Score 1, 2	119	31	150
	Score 3, 4, 5	18	2	20
**P. nigrescens**	Score 1, 2	44	12	56
	Score 3, 4, 5	28	6	34
**A. actinomycetemcomitans**	Score 1, 2	50	8	58
	Score 3, 4, 5	6	2	8
**C. rectus**	Score 1, 2	56	14	70
	Score 3, 4, 5	3	1	4
**T. denticola**	Score 1, 2	66	19	85
	Score 3, 4, 5	3	1	4

**Table 2:** Comparison of HP and NRAMP1-rs2276631 alleles regarding the frequencies of iron-dependent bacteria

**HPandrs2276631**		**Allele 2, G**	**Allele 1, A**	**Total**
**P. gingivalis**	Score 1, 2	115	35	150
	Score 3, 4, 5	30	6	36
**P. intermedia**	Score 1, 2	116	34	150
	Score 3, 4, 5	16	4	20
**P. nigrescens**	Score 1, 2	42	14	56
	Score 3, 4, 5	26	8	34
**A. actinomycetemcomitans**	Score 1, 2	46	12	58
	Score 3, 4, 5	6	2	8
**C. rectus**	Score 1, 2	51	19	70
	Score 3, 4, 5	3	1	4
**T. denticola**	Score 1, 2	67	19	86
	Score 3, 4, 5	3	1	4

**Table 3:** Frequency of iron-dependent bacteria in HP, rs1723540, and rs2276631

**rs2276631andrs1723540andHP**		**Allele 2, G**	**Allele 1, A**	**Total**
**P. gingivalis**	Score 1, 2	187	39	226
	Score 3, 4, 5	47	7	54
**P. intermedia**	Score 1, 2	187	39	226
	Score 3, 4, 5	26	4	30
**P. nigrescens**	Score 1, 2	70	14	84
	Score 3, 4, 5	42	10	52
**A. actinomycetemcomitans**	Score 1, 2	75	13	88
	Score 3, 4, 5	10	2	12
**C. rectus**	Score 1, 2	85	21	106
	Score 3, 4, 5	5	1	6
**T. denticola**	Score 1, 2	106	23	129
	Score 3, 4, 5	5	1	6

Table 1 compares the frequencies of iron-dependent bacteria in HP and NRAMP1-rs1723540 alleles. As shown, a *P. gingivalis* score of 1–2 was 4.5 times higher in individuals carrying 2 and G alleles compared with 1 and A alleles. Also, a *P. gingivalis* score of 3–5 was 5 times more common in HP 2 and rs1723540 G alleles. A *P. intermedia* score of 1–2 was 3.8 times higher in 2 and G alleles compared with 1 and A alleles. In addition, a *P. intermedia* score of 3–5 was 9 times more prevalent in HP 2 and rs1723540 G alleles. A *P. nigrescens* score of 1–2 was 3.6 times more frequent in 2 and G alleles. A *P. nigrescens* score of 3–5 was 4.6 times more common in HP 2 and rs1723540 G alleles compared with 1 and G alleles. An *A. actinomycetemcomitans*score of 1-2 was 6.2 times higher in HP 2 and rs1723540 G alleles. An *A. actinomycetemcomitans*score of 3–5 was 3 times more frequent in 2 and G alleles. A *C. rectus* score of 1–2 was 4 times higher in HP 2 and rs1723540 G alleles. Additionally, a *C. rectus* score of 3–5 was 3 times more common in 2 and G alleles. A *T. denticola* score of 1-2 was 3.5 times higher in HP 2 and rs1723540 G alleles. A *T. denticola* score of 3–5 was 3 times more common in 2 and G alleles.

Table 2 compares the HP and NRAMP1-rs2276631 alleles regarding the frequencies of iron-dependent bacteria. As shown in the table, a *P. gingivalis* score of 1–2 was 3.3 times higher in individuals carrying HP 2 and rs2276631 G alleles compared with HP 1 and rs2276631 A alleles. A *P. gingivalis* score of 3–5 was 5 times more common in 2 and G alleles. A *P. intermedia* score of 1–2 was 3.4 times more common in HP 2 and rs2276631 G alleles. A *P. intermedia* score of 3–5 was 4 times higher in 2 and G alleles. A *P. nigrescens* score of 1-2 was 3 times more prevalent in HP 2 and rs2276631 G alleles. Also, a *P. nigrescens*score of 3–5 was 3.2 times higher in 2 and G alleles. An *A. actinomycetemcomitans*score of 1–2 was 3.8 times higher in HP 2 and rs2276631 G alleles. Also, an *A. actinomycetemcomitans*score of 3–5 was 3 times more frequent in 2 and G alleles. A *C. rectus* score of 1–2 was 2.7 times more common in HP 2 and rs2276631 G alleles. A *C. rectus* score of 3–5 was 3 times higher in 2 and G alleles. A *T. denticola* score 1-2 was 3.5 times more common in HP 2 and rs2276631 G alleles. *T. denticola* score of 3–5 was 3 times more frequent in 2 and G alleles.

Table 3 presents the frequencies of iron-dependent bacteria in HP, rs1723540, and rs2276631. This combined allele analysis showed the frequencies of bacteria when individuals had the weakest bond between iron and HP/NRAMP1 (rs1723540+ rs2276631). A *P. gingivalis* score of 1–2 was 4.8 times higher in people carrying HP 2, rs1723540 G, and rs2276631 G alleles compared with HP 1, rs1723540 A and rs2276631 A alleles. A *P. gingivalis* score of 3–5 was 6.7 times more common in 2 and both G alleles. A *P. intermedia* score of 1–2 was 4.8 times more frequent in HP 2, rs1723540 G, and rs2276631 G alleles. A *P. intermedia* score of 3–5 was 6.5 times more common in 2 and both G alleles. A *P. nigrescens* score of 1–2 was 5 times higher in HP 2, rs1723540 G, and rs2276631 G alleles. A *P. nigrescens* score of 3–5 was 4.2 times more common in 2 and both G alleles. An *A. actinomycetemcomitans*score of 1–2 was 5.8 times more frequent in HP 2, rs1723540 G, and rs2276631 G alleles. An *A. actinomycetemcomitans*score of 3–5 was 5 times higher in 2 and both G alleles. A *C. rectus* score of 1–2 was 4 times more common in HP 2, rs1723540 G, and rs2276631 G alleles. A *C. rectus* score of 3–5 was 5 times more frequent in 2 and both G alleles. A *T. denticola* score of 1–2 was 4.6 times more common in HP 2, rs1723540 G, and rs2276631 G alleles. A *T. denticola* score of 3–5 was 5 times more prevalent in 2 and both G alleles.

## Discussion

Chronic periodontitis and peri-implantitis are characterized by the presence of bacteria, inflammation, and bone loss around the teeth and implants, respectively. Some of these pathogenic bacteria consume heme. The primary natural source of heme is probably the host organism, but the human circulatory system contains various heme-sequestering proteins, such as HP and NRAMP1, which are also present in high concentrations in the gingival crevicular fluid.^25^ Therefore, HP and NRAMP1 might have some protective effects on periodontal tissues by binding to Hb and limiting the growth and proliferation of pathogenic bacteria.

The diversity in HP phenotypes causes different binding affinities for Hb (HP1-1>HP1-2>HP2-2).^26^ Also, some polymorphisms of the NRAMP1 gene (rs1723540 and rs2276631 A to G polymorphisms) can change its function and decrease its binding affinity for Hb.

This study aimed to evaluate our hypothesis that SNPs in the HP and NRAMP1 gene could alter the accumulation of periopathogenic microorganisms by changing the iron concentration (free Hb). On the other hand, we aimed to assess whether HP/NRAMP1 SNPs can have bacteriostatic activity in vivo.

The current results offered a new perspective on periodontal and peri-implant pathogenesis. The selected periopathogenic microorganisms were more frequent in patients carrying HP 2 and both NRAMP1 G alleles. This finding revealed that HP and NRAMP1 might have bacteriostatic activity in vivo.

To the best of the authors’ knowledge, the relationship between HP and NRAMP1 allele frequencies and the presence of heme-consuming periodontal pathogens has not been previously evaluated; thus, more evidence is required to reach a valid conclusion in this respect.

Nibali et al^27^ (2007) found that the Fc gamma receptor and IL-6 polymorphisms were associated with increased counts of *A. actinomycetemcomitans*, *P. gingivalis*, and *T. forsythia*. Holla et al^3^ (2011) reported no significant association between the interferon gamma polymorphism and the presence of subgingival periodontal pathogens.

The DNA checkerboard hybridization technique applied in this study has several advantages. This technique is fast, sensitive, and relatively inexpensive, and provides quantitative data. It overcomes many of the limitations of culture-based microbiology, including the loss of viability of organisms during transport.^16,17^

## Conclusion

Within the limitations of this study, it seems that there is a relationship between HP and NRAMP1 allele frequencies and the presence of heme-consuming periodontal pathogens in the Iranian patients with chronic periodontitis and peri-implantitis. However, this finding should be interpreted with caution due to the small sample size of the study. Further investigations with larger sample sizes on different populations are required to confirm the current findings.

## Authors’ Contributions

Design: MK and ARE designed the study. RA and ZAT collected the data. ZAT drafted the primary manuscript. All authors have read and approved the final manuscript.

## Competing Interests

The authors declare that they have no competing interests.
